# Inverted Topologies in Membrane Proteins: A Mini-Review

**DOI:** 10.5936/csbj.201308004

**Published:** 2013-11-09

**Authors:** Amanda M Duran, Jens Meiler

**Affiliations:** a Center for Structural Biology, Department of Chemistry, Vanderbilt University, Nashville, TN 37212, USA

**Keywords:** Membrane protein, symmetry, inverted topology, gene duplication, gene fusion, pseudo-symmetry

## Abstract

Helical membrane proteins such as transporters, receptors, or channels often exhibit structural symmetry. Symmetry is perfect in homo-oligomers consisting of two or more copies of the same protein chain. Intriguingly, in single chain membrane proteins, often internal pseudo-symmetry is observed, in particular in transporters and channels. In several cases single chain proteins with pseudo-symmetry exist, that share the fold with homo-oligomers suggesting evolutionary pathways that involve gene duplication and fusion. It has been hypothesized that such evolutionary pathways allow for the rapid development of large proteins with novel functionality. At the same time symmetry can be leveraged to recognize highly symmetric substrates such as ions. Here we review helical transporter proteins with an inverted two-fold pseudo-symmetry. In this special scenario the symmetry axis lies in the membrane plane. As a result, the putative ancestral monomeric protein would insert in both directions into the membrane and its open-to-the-inside and open-to-the-outside conformations would be structurally identical and iso-energetic, giving a possible evolutionary pathway to create a transporter protein that needs to flip between the two states.

## Pseudo symmetry in soluble proteins

**Figure 1:**
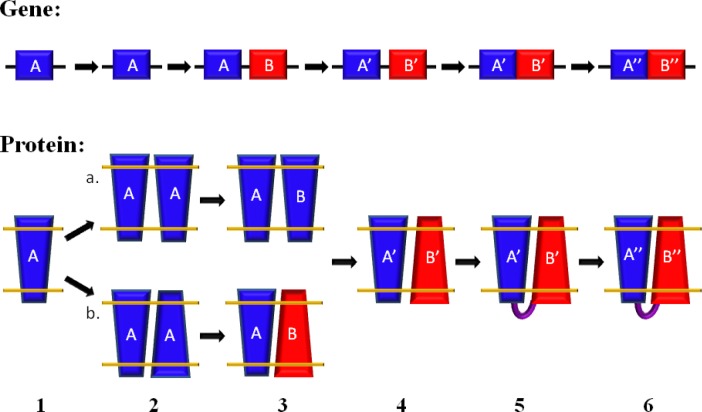
Proposed evolutionary pathway for membrane proteins with inverted symmetry involving the gene duplication and fusion hypothesis. Step 1. Prior to a gene duplication event, gene A exists as a singular gene. Step 2. The translation product of the gene, protein A, has an odd number of trans-membrane spans, and has a preferred orientation (no dual topology, 2a) or is attracted to itself and exhibits dual-topology (2b). Step 3. A gene duplication event occurs to produce sequence identical genes A and B, which are composed of the same sequence (3ab). Step 4. Both gene A and B acquire mutations independently of each other resulting in genes A’ and B’. For path a, mutations cause a switched in protein's B bias to insert into the membrane resulting in proteins of opposite topology. For path b, this means mutations have stabilized each protein in its respective topology. Step 5. Related genes A’ and B’ undergo a gene fusion event and are connected by a loop (green). Step 6. Additional mutations cause further sequence divergence resulting in a protein with homologous subunits A’’ and B’’.

In the realm of soluble proteins, ten folds are over-represented and dominate the structures determined so far experimentally in the Protein Data Bank (PDB) [1]. Such common ‘superfolds’ in proteins likely exist because nature evolved existent protein folds as opposed to generating new folds [2]. Six of these ten superfolds display pseudo-symmetry, i.e. can be seen as a repeat of usually two or more copies of nearly identical structural subunits. These folds are: Ferredoxin fold, β-trefoil, up-down bundle, immunoglobulin fold, jelly-roll, and the TIM-barrel fold [3]. The TIM-barrel fold is a repeat of eight β-strand-α-helix units where the eight β-strands form an inner barrel surrounded by the eight α-helices. Close inspection of the hydrogen bonding pattern in the barrel reveals that the fold is a 4-fold symmetric arrangement of β-strand-α-helix-β-strand-α-helix units [3]. Many enzymes share this (βαβα)_4_ fold some recognizing pseudo-symmetric substrates. Similarly, four-helix bundles with C2 and C4 symmetry are commonly seen as homo-dimers and homo-tetramers [3]. It has been postulated that symmetry at the fold level evolved via gene duplication and fusion events from homo-oligomeric proteins [4, 5] (Figure 1). Fusion of monomer units into a single domain increases thermodynamic stability and kinetic foldability [6]. Gene duplication is thought to relieve selective pressure which allows for diversification of the subunits on the sequence level before and/or after the fusion event (Figure 1) to achieve more complex biological functions [3]. As different mutations occur in the two copies of the gene, the evidence of symmetry is masked at the level of the primary sequence. It is assumed that this strategy is one route to evolve large proteins with complex functions rapidly in nature. At the same time symmetry is explored as an avenue for rational or computational design of large protein domains [7, 8].

## Self-attraction and self-association of protomers

**Figure 2:**
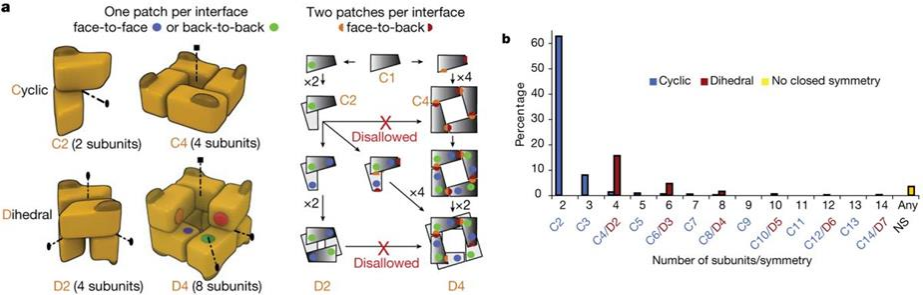
Assembly of protomers into oligomers. Assembly can be organized in a cyclic or dihedral manner. Symmetry axes are represented by the dotted lines where two-fold are labeled with ellipses and four-fold are labeled with squares. Cyclic arrangement allows for face-to-back contacts between protomers while dihedral arrangement allows for additional interface contacts between protomers (2a). Cyclic assembly is the overall most common type of arrangement; however, dihedral is common in tetramers (2b). Reprinted by permission from Macmillan Publishers Ltd: *Nature*, advance online publication, 18 June 2008 (doi: 10.1038/sj.*Nature*.06942)

For gene duplication and fusion as a viable strategy to create large protein domains, interaction of a protein with itself, self-attraction, is a prerequisite. And indeed, homo-oligomers are abundant in the Protein Data Bank (PDB). Homo-oligomers are more stable and therefore more prevalent, as they tend to have a lower energy than their hetero-oligmeric counterparts [9]. There are two basic ways in which a protein can be attracted to itself. The first type of self-association is where the same faces of the protein are attracted to each other and form the dimerization interface. The remaining faces are left and can interact with similar remaining faces to form larger oligomers. The second, less common form of self-association occurs when two different faces are attracted to each other. This creates a cyclic oligomeric structure [10] (Figure 2).

Interestingly, the majority of homo-dimeric complexes in the PDB exhibit a symmetric arrangement of the two protomer units. In this arrangement all interactions between the two protomers are duplicated which halves the total number of unique interactions that are possible. This causes a bias towards very-low-energy symmetric homo-dimeric complexes. With one patch of the protein interacting with the same patch of another copy, such arrangements are evolutionarily stuck in dimeric symmetry as continued evolution into homo-oligomers with higher-order cyclic symmetry requires interaction of two distinct patches (Figure 2).

Nevertheless, cyclic symmetry while less frequent is still observed on the homo-oligomeric level. Starting from these cyclic homo-oligomeric proteins internal cyclic symmetry can evolve via gene duplication and fusion. The TIM-barrel and β-propeller superfolds are prominent examples [3]. However, applying the gene duplication and fusion hypotheses to the study of membrane protein evolution has proven difficult due to sparseness of membrane protein structures.

## Sparseness of membrane protein structures complicate determination of evolutionary pathways

One of the biggest limiting factors in studying membrane protein topology and symmetry is the small number of membrane protein structures that have been determined [11]. Currently, only 289 unique helical membrane protein structures are available [12]. These represent only about 120 distinct folds i.e. structurally distinct arrangements of two or more trans-membrane helices. On the other hand, analysis of sequence databases reveals 1,200 families of proteins with more than one predicted trans-membrane helix. These families are distinct in the sense that no inter-family homology can be detected on the sequence level [13]. While some of these families might turn out to share a fold on the structural level, this result also implies that many membrane proteins of unknown topology remain to be determined. During the past five years between five and ten novel membrane protein topologies have been determined per year. However, many more structures will need to be determined before the evolutionary pathways are better supported and understood.

## Internal repeat symmetry in monomeric membrane proteins

Symmetry in proteins can improve stability and aids in overcoming energy hurdles in conformational change pathways [6, 14]. In some cases internal repeat symmetry (IRS) can be detected by sequence analysis. However, because the sequence of membrane proteins evolves quickly, IRS is often only confirmed after the structure of the protein has been determined [15, 16]. IRS is hypothesized to originate from gene duplication events or by fusion of similar subunits [15]. In a 2007 study by Choi and coworkers, it was found that almost half of known α-helical membrane proteins have internal repeat symmetry. Types of symmetry include n-fold rotational or cyclic symmetry and inverted symmetry. As the symmetry is only present at the structural level but not at the sequence level it is often referred to as pseudo-symmetry [17].

## The lipid environment restricts the fold space for membrane proteins

**Figure 3:**
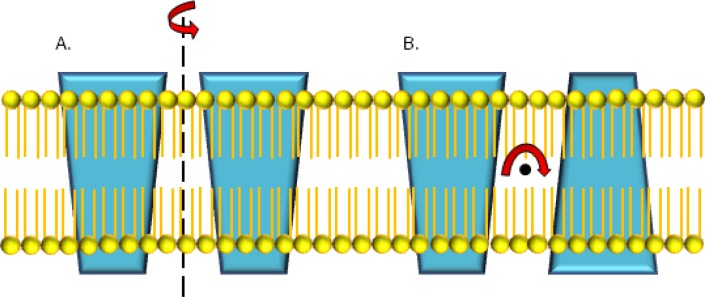
Symmetry axes for membrane proteins. The rotational symmetry axis can either be parallel to the membrane normal and orthogonal to the membrane plane (3a). The axis can also be orthogonal to the membrane normal and parallel to the membrane plane. When rotated 180° along this axis, the resulting structure will resemble the starting structure.

For membrane proteins, the lipid environment restricts conformation[18]. Along with symmetry, and self-association, these observations have a number of important consequences for membrane protein topology: homo-dimeric proteins with a symmetric arrangement of the two protomer units can align their symmetry axis either parallel or orthogonal to the membrane normal [19] (Figure 3). Higher-order (larger than two) homo-oligomers with cyclic symmetry can only embed into the membrane with the symmetry axis parallel to the membrane normal, i.e. orthogonal to the two-fold symmetry axis of the membrane in the membrane plane. Any other arrangement would break the symmetry in the homo-oligomer. In consequence, we observe two major classes of homo-oligomeric membrane proteins and resulting pseudo-symmetric membrane proteins when considering alignment with respect to the membrane.

### (1) Symmetry axis parallel to membrane normal and orthogonal to membrane plane

Proteins embedded in the outer membrane often form β-barrels. These can be seen as cyclic repeats typically consisting of 3-10 ββ-hairpins with the pseudo-symmetry axis parallel to the membrane normal [20]. β-barrel monomers are also known to assemble into higher order oligomers. For example, cholesterol-dependent cytolysins are capable of forming aqueous pores that consist of up to fifty monomers [21]. However, approximately 70% of the unique membrane protein structures are α-helical including receptors, transporters, and channels [12]. A variety of homo-oligomeric and pseudo-symmetric proteins are observed with the symmetry axis parallel to the membrane normal. For example, the single trans-membrane span glycophorin [22] forms a homo-dimer, diacylglycerol kinase [23] forms a homo-trimer, voltage-gated potassium channel [24] forms a homo-tetramer, and several eukaryotic ABC transporters such as TM287/288 [25] form a hetero-dimer. Note, that all N- and C-termini of the protomers will always assemble on the same side of the membrane, i.e. the protomers insert into the membrane in the same direction. These homo-oligomers typically follow the positive-inside rule which states that positively charged residues Arginine and Lysine tend to face towards the inner leaflet of the membrane [26]. Most membrane proteins have such a well-defined orientation based on the distribution of positively charged residues. Resulting homo-oligomers have C2, C3, or C4 symmetry with the rotational axis of symmetry parallel to the membrane normal [8, 19]. For example, single chain voltage gated sodium channels exist in humans that resemble the homo-tetrameric structure of the bacterial voltage-gated sodium channel NavAB [27]. For these proteins to evolve into a single chain, monomeric membrane proteins require an even number of trans-membrane spans to satisfy the gene duplication and fusion hypothesis.

### (2) Symmetry axis orthogonal to membrane normal and parallel to membrane plane

In the inverted symmetry scenario, protomers insert into the membrane in opposite directions [19]. This arrangement is only feasible for homo-dimers as for higher-order oligomers the symmetry would be broken by the 2-fold symmetry of the membrane when ignoring the differences between inner and outer leaflet in natural membranes. N- and C-termini of the protomers are on opposite sides of the membrane, respectively. Examples of proteins with inverted pseudo-symmetry are the glycerol facilitator channel [15, 28, 29], the leucine transporter [30], and the urea transporter [31]. They contain an odd number of helices in the symmetric unit. In some cases half helices or re-entrance loops are observed which will meet with its symmetric counterpart at the middle of the membrane. An odd number of trans-membrane spans is required for the gene duplication and fusion hypothesis to be a possible evolutionary route to pseudo-symmetric monomeric proteins.

## Effect of inverted symmetry on transporter proteins with open-to-the-inside and -outside conformations

Sequence conserved regions of proteins are referred to as internal repeat cores (IRC) and are typically found at the symmetric interface. It has been proposed that this region is the most conserved because of the self-attractive interactions needed in stabilizing the two symmetric subunits and the role it has in the two-state conformational switch for the inactive and active transport of molecules [15]. Interestingly, inverted pseudo-symmetry is particularly frequent in transporter proteins which can be explained with the necessity of having at least an open-to-the-inside and an open-to-the-outside conformation in an alternate access mechanism of transport. For example, LeuT has an inverted internal repeat of five trans-membrane helices. The inverted structural symmetry inherently creates a channel with a symmetric pathway across the membrane because the structurally symmetric units are placed opposite of each other. The perfectly symmetric structure can be leveraged to create structurally identical and iso-energetic inward and outward facing conformations so that no major energy barriers would need to be overcome to transport substrate across the membrane. As a transporter, the symmetric pathway helps form inward and outward conformations [32]. With core functional residues conserved, chemically similar residues and structures are on either side of the membrane, enabling bidirectional transport of molecules across the membrane [15].

## Sparseness of membrane protein homo-dimers with inverted symmetry

Despite the abundance of membrane proteins with inverted two-fold pseudo-symmetry, homo-dimers with inverted symmetry seem to be rare. Formation of these requires “dual topology”, i.e. the ability for a single subunit to exist in both orientations in the same membrane and environmental conditions [19, 33]. The existence of proteins capable of dual topology is heavily debated, with one of the best studied examples being the homo-dimeric efflux-multidrug transporter from *Escherichia coli* (*E. coli*), EmrE [19]. A recent NMR study suggests that EmrE is able to exist in either orientation as both states are energetically similar [34]. EmrE with an even number of trans-membrane spans cannot readily undergo gene duplication and fusion, i.e. it is evolutionarily frustrated.

In 2006, Rapp *et al*. proposed five proteins that have potential as proteins capable of dual topology. These proteins are small, composed of four trans-membrane spanning helices, and have very little positive charge bias [5]. It makes sense that a protein with dual topology would be small to act as a unit of symmetry and have very little positive charge bias to readily be placed in either orientation in the membrane without disobeying the positive-inside rule [26]. Additionally, an overall neutral charge causes both topologies to be similar in energy [17, 35]. To further understand the significance of a negligible positive charge bias in dual topology, membrane proteins with a positive charge bias of nearly zero were engineered to have a distinct bias. The engineered bias caused a flip in orientation for these proteins [5]. In an evolutionary route over time, mutations to a fused dual-topology protein could essentially lock in a particular topology while maintaining functionally important residues.

## Dual topology is not required for evolution of membrane proteins with inverted two-fold pseudo-symmetry

The apparent sparseness of homo-dimers with inverted symmetry seems to be at odds with the abundance of membrane proteins with inverted two-fold pseudo-symmetry. However, it is important to note that a homo-dimer with inverted symmetry is not a prerequisite for the evolution of a membrane protein with inverted two-fold pseudo-symmetry. Consider the following putative evolutionary pathway (Figure 1): a membrane protein gene with preferred orientation in the membrane gets duplicated. In one copy mutations occur that change the preferred orientation within the membrane. An interaction between the two proteins evolves that because of their similarity is still likely to be pseudo-symmetric. At this time the protein develops its transport functionality. A gene fusion event creates the inverted two-fold pseudo-symmetric protein.

In this context a 2006 study by Rapp *et al*. used *E. coli* membrane proteins and anti-parallel hetero-dimer pair YdgE and YdgF as examples of homologous proteins with different positive charge biases and opposite orientations. *E. coli* proteins YdgE and YdgF are overlapping genes on the chromosome, but are expressed separately [5]. YdgE is known to consist of four trans-membrane spans whereas YdgF is predicted to consist of four [36]. YdgQ and YdgL are another example of a homologous gene pair in *E. coli* that results in proteins with opposite orientations. For both of these pairs of proteins, each protein has a positive charge bias favoring its respective orientation [5]. Because of the opposing orientations, each homologous pair is able to form an anti-parallel hetero-dimer. These anti-parallel hetero-dimers are likely the result of gene duplication and topology evolution events [37].

Rapp *et al*. suggested five dual topology possibilities (Table 1). Two pairs of homologous hetero-dimers which form anti-parallel topologies are also included in this table. Positive charge bias was calculated similarly to Rapp *et al*. where counts of K and R in the even loops are subtracted from the odd loops, where the N-terminal loop is loop 1.

**Table 1:** Dual topology and anti-parallel hetero-dimer candidates.

Protein	Predicted Topology	TM Spans	Positive Charge Bias
EmrE	Dual	4	-2
SugE	Dual	4	-1
CrcB	Dual	4	0
YdgC	Dual	3	1
YnfA	Dual	4	0
YdgE	Anti-parallel hetero-dimer	4	7
YdgF	Anti-parallel hetero-dimer	4	6
YdgQ	Anti-parallel hetero-dimer	05-06	-6
YdgL	Anti-parallel hetero-dimer	4	-7

## Gene duplication and fusion as it applies to monomeric membrane proteins

In a 2008 study, Lolkema *et al*. studied the DUF606 family to get clues for the possible order of evolutionary events. In the DUF606 family, there exist homo-dimeric proteins, hetero-dimeric proteins, and two-domain fusion proteins which are proposed to be indicative of single gene dual topology proteins, homologues of opposite orientations, and fused genes creating an anti-parallel topology, respectively [37]. They found no existing fused homo-dimeric protein in the DUF606 family as evidence for direct fusion of duplicated genes. The evolutionary pathway that was proposed as a result of this study involved a gene duplication event followed by sequence divergence and finally a gene fusion event between the homologues.

Previously, it has been suggested that one of the likely evolutionary routes begins with gene duplication shortly followed by gene fusion. Following fusion, divergence further stabilizes the energetics, anti-parallel topology, and function [19]. However, evidence of any fused homo-dimer have not yet been found [37]. Yet, extensive divergence prior to a fusion event would seem to affect the self-attraction between the two domains. Therefore, in Figure 1, we propose a slightly modified version of the alternative evolutionary route for inverted membrane protein topologies. First, the gene capable of dual topology is duplicated by the appropriate evolutionary mechanism, a gene duplication event. Next, the domains of the homo-dimers are stabilized into opposite orientations by mutations which stabilize the overall anti-parallel topology. Then, the similar domains undergo fusion followed by even further sequence divergence to stabilize structure and improve function. However, there is currently insufficient evidence to support one route over the other.

## Major Facilitator Superfamily

The major facilitator superfamily (MFS) transporters have been extensively studied for their symmetry. Proteins in the MFS transporter family are composed of 12 trans-membrane spans. The sequence homology between other members of this family is weak; however, proteins in this family are structurally similar [38]. There have been differences in opinion for the breakdown in symmetry. In 2012, a review proposed that the smallest symmetric unit is a two trans-membrane spanning domain [38]. This would mean that there is three-fold symmetry within the six helix bundles and then an additional two fold inverted symmetry for the six helix bundles. However, previous studies have supported the idea of a three trans-membrane spanning structural motif resulting in two-fold symmetry in the 6 helix bundle [39]. Recently in 2013, Madej *et al*. conducted an experiment where the symmetry motifs in MFS protein L-fucose H+ symport protein FucP were rearranged [40]. The result was a structure strikingly similar to LacY, another member of the MFS. The conclusion was that FucP and LacY likely evolved from the same primordial helix triplets, but the order of assembly of these structural motifs into larger proteins differed which created an avenue for diversity in function [40].

## Neurotransmitter Sodium Symporters

Neurotransmitter sodium symporters are also a type of transporter proteins which display internal symmetry. The most well-known examples display two-fold pseudo-symmetry and include the glutamate transporter (GltPh), the sodium and proton antiporter (NhaA), and the leucine transporter (LeuT) [41]. A rocker switch mechanism of transport favors the internal two-fold symmetry because the conformation is easily exchanged [32, 41]. In LeuT, the trans-membrane spans 1-5 are symmetric to 6-10. LeuT can be considered an occluded state that can convert into outward and inward facing conformations due to the internal symmetry. Furthermore, the addition of non-symmetric helices act as hinges to promote conformational change during transport [17, 32].

## Aquaporins

The aquaporins are another type of membrane protein that exhibit pseudo-symmetry. In fact, the very first high resolution example of inverted topology was from *E. coli*'s aquaglyceroporin the glycerol facilitator protein (GlpF) [42]. Aquaporins are a great example of symmetry observed on a single polypeptide chain. They are made up of six trans-membrane spanning helices and two half-spanning helices with the symmetric unit being three and a half helices. The α carbon root mean square deviation between the two halves of GlpF is 1.8 Angstroms [15]. Channel proteins’ primary function is the transport of water and small molecules across the membrane. Inverted symmetry is advantageous for the formation of a symmetric pathway across the channel [32]. However, because transport through the channel is permeation instead of a two-switch conformational change, the advantage of inverted symmetry is largely for stability of the protein. For this reason, channels are sometimes referred to as broken transporters [32]. GlpF and other aquaporins have an aspartic acid-proline-alanine motif seen in both halves at the symmetric interface [15, 28, 43]. In this example, stability is improved because of the interaction between the proline rings on either half [28].

## Chloride Channel

Another type of channel protein that exhibits symmetry is the ClC chloride channel [42, 44]. A single subunit in the homo-dimeric complex is made up of 18 helices. Eight helices on the N-terminal half display striking inverted two-fold pseudo-symmetry with the C-terminal half. Like the aquaporins, the anti-parallel structure is useful for this channel protein because the symmetric polar ends of helices are able to face the outside of the membrane. This is energetically favorable in that the polar ends are not buried in the membrane [44]. Interestingly, another ion channel, the potassium channel, does not take advantage of anti-parallel topology. The potassium channel works very differently in that the cavity widens near the center of the membrane. The helix dipoles are also positioned very differently, in a parallel fashion, to help overcome the dielectric barrier which is the nature of the membrane. However, in this anion channel, the anti-parallel topology creates a selectivity filter for chloride ions. It is predicted that the reason for this vast difference in topology is because hydrophobic anions partition into membranes much more readily than hydrophobic cations, so channels transporting cations would need a much larger cavity to stabilize the cation [44].

## Effect of lipid composition of membrane protein topology

A factor largely ignored in this review is lipid composition and differences between inner and outer leaflet of the membrane [45]. In 2013, Vitrac and colleagues found that when the composition of phosphatidylethanolamine (PE) was varied in a lipid environment, proteins were capable of complete inverted topology. Native and inverted conformations of lactose permease (LacY) from *E. coli* were found to exist in the membrane at the same time. Thermodynamically, dual topology is partially determined based off of the inherent properties of the protein interaction with the lipids in the membrane. Studies both *in vitro* [45, 46] and *in vivo* [47] show that through the manipulation of protein domain charge or lipid composition, dual topological arrangements of a protein can co-exist in the same membrane. It is important to keep in mind that membrane proteins not only evolve with time but in concert with lipid environments that can also affect topology between homologous proteins.

## Overcoming insufficient structural information

Many cases of internal repeat symmetry in membrane protein have been difficult to recognize until after structure determination [15, 16]. Often times, as shown in many of the aforementioned cases, the sequence identity is low because of such extensive sequence divergence despite maintaining structural symmetry. Because it is not feasible to determine the structure for all proteins of interest in order to detect symmetry, other physical properties have been employed to provide additional information towards the prediction of internal symmetry. In particular, hydropathy profiles have recently been used to detect internal symmetry of transporters [16]. Instead of looking at raw sequence similarity, AlignMe [16, 48] takes into consideration the hydrophobicities of amino acids as a tool for alignment. The advantage is that physical properties like hydrophobicites will be more conserved over time and will match proteins that resemble each other chemically. This can improve the ability to detect internal symmetries where structural information is unavailable.

**Figure 4:**
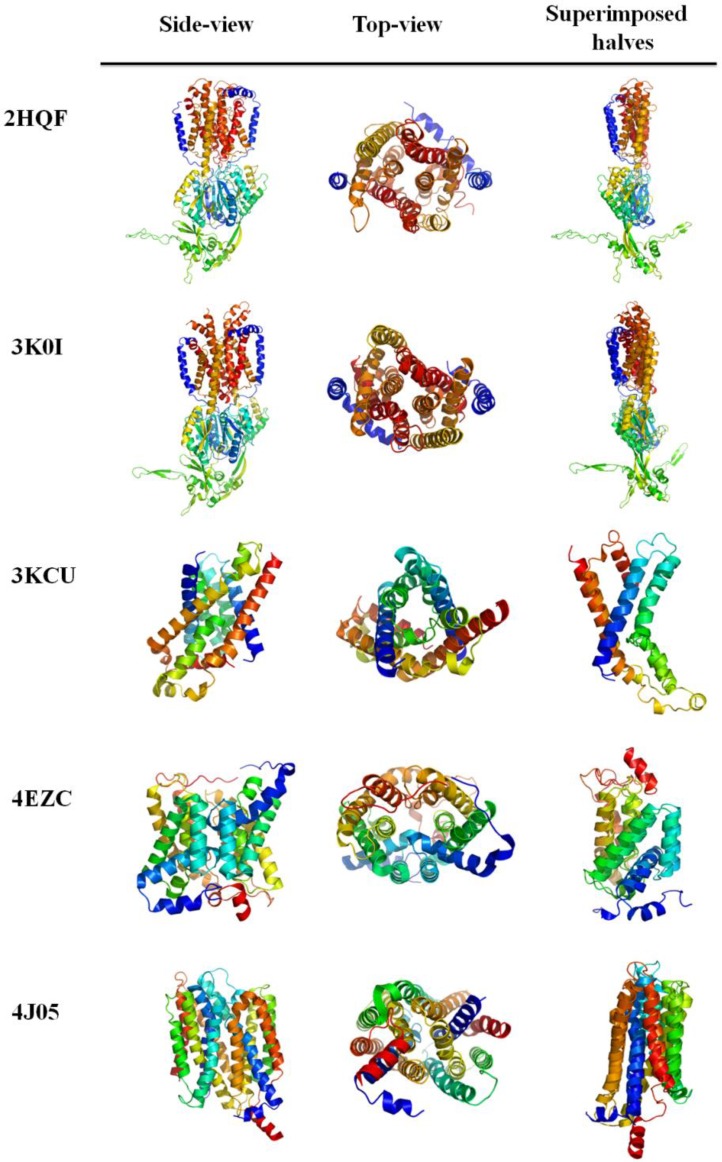
Superimposition of pseudo-symmetric halves. Five symmetric membrane proteins since 2007 are shown on the left as a monomer. The middle shows a view of the symmetry from the top. On the right, the pseudo-symmetric halves are superimposed to show the striking structural similarity. Cα RMSD for these proteins can be found in Table 2.

The most obvious limiting factor in understanding more about membrane protein evolution and pseudo-symmetry is the limited number of known membrane protein structures. Table 2 displays proteins with detected internal symmetry in Choi *et al* [15]. Additional, selected membrane protein structures determined since 2007 were added, when symmetry was obvious. For these, we calculated Cα RMSD for the trans-membrane spanning helices using PyMol [49] software. The OCTOPUS [50] server was used to determine the location of the loops with respect to the membrane. Here, positive charge bias was calculated by the number of “inside” K and R residues minus the number of “outside” K and R residues. In Figure 4, six of these structures were chosen to visualize the symmetry from both side and top views with corresponding trans-membrane helices colored accordingly.

**Table 2:** Internal Symmetry in select membrane protein structures.

Protein Name	PDB ID	#TM spans/unit	% Identity	Cα RMSD	Symmetry Type	Membrane Symmetry Axis	Positive Charge Bias
Cytochrome C Oxidase	1OCC^a^	4	15.6	3	C3	Normal	6
Formate dehydrogenase-N	1KQF^a^	2	18.1	3.8	C2	Normal	21
Mitochondrial ADP/ATP carrier	10KC^a^	2	23.5	1.8	C3	Normal	16
Rotor of V-type Na ATPase	2BL2^a^	2	28.9	1.2	C2	Normal	5
Spinach photosystem II	1RWT^a^	1	23.1	3.3	C2	Normal	6
BtuCD vit B13 transporter	1L7V^a^	2.5	21.5	3.4	Inverted C2	Plane	7
Bovine rhodopsin	1U19^a^	3	17.1	4.7	C2	Normal	11
Archaerhodopsin-2	1VGO^a^	3	9.2	4.4	C2	Normal	7
AQP1 water channel	1J4N^a^	3.5	17.6	2.5	Inverted C2	Plane	10
Glycerol Facilitator channel	1FX8^a^	3.5	18.5	1.8	Inverted C2	Plane	5
H/Cl exchange transporter	1KPK^a^	5	17.9	2.7	Inverted C2	Plane	23
Amt-I ammonia transporter	2B2F^a^	4.5	11.8	2.3	Inverted C2	Plane	12
LeuTAa leucine transporter	2A65^a^	2.5	17.8	4.5	Inverted C2	Plane	9
AcrB bacterial multi-drug efflux transporter	1IWG^a^	5	16.4	2.1	C2	Normal	16
Nha Na/H antiporter	1ZCD^a^	3	19.5	3.3	Inverted C2	Normal	11
CusA transporter	3K0I	5	21.9 (360 Residues)	3.46	C2	Normal	12
AcrB bacterial multi-drug efflux transporter	2HQF	3	17.9 (218 Residues)	3.679	C2	Normal	15
Phosphate Transporter	4J05	6	21.4 (131 Residues)	3.794	C2	Normal	2
Formate Channel	3KCU	3.5	34.6 (122 Residues)	5.611	Inverted C2	Plane	5
Urea Transporter	4EZC	5.5	27.9 (147 Residues)	1.926	Inverted C2	Plane	8

a Membrane proteins from Choi et al 2007

In summary, inverted topology in membrane proteins could have evolved via multiple evolutionary routes. While symmetric self-association is known as a stabilizing factor for protein structure, inverted topology within membrane proteins adds an interesting twist to the puzzle as it implies dual topology membrane proteins, i.e. proteins that can insert into the membrane in both directions. However, it is also possible that attraction between the two protomers only evolved after gene duplication and after one copy of the gene underwent mutations that inverted its topology. Such symmetric interactions between almost identical proteins would still be energetically favorable as many residues in the interface would adhere to the symmetry condition. With insufficient evidence to prefer one route over the other, efforts continue to understand how inverted symmetry in membrane proteins evolved.
